# Pesticides application rate maps in the European Union at a 250 m spatial resolution

**DOI:** 10.1038/s41597-025-05031-7

**Published:** 2025-05-01

**Authors:** G. M. Porta, L. Casse, A. Manzoni, M. Riva, F. Maggi, A. Guadagnini

**Affiliations:** 1 Department of Civil and Environmental Engineering, Politecnico di Milano, Piazza Leonardo da Vinci 32, 20133 Milano, Italy; 2 Environmental Engineering, School of Civil Engineering, The University of Sydney, Sydney, New South Wales Australia

**Keywords:** Environmental impact, Hydrology, Agriculture

## Abstract

Our work targets mapping of pesticides application rates within the European Union at a 250 m spatial resolution. Source data include global estimates of pesticide inputs, high resolution crop maps and pesticide usage reported by EUROSTAT official figures. Previously published global pesticide application rates in PEST-CHEMGRIDS are used as first guess estimates. These are then adjusted using a calibration dataset gathered from pesticide use in agriculture. The estimation of the applied mass by country and crop type is then combined with high resolution crop maps. The procedure explicitly accounts for data quality and uncertainty through a Maximum Likelihood estimation procedure. This data product features detailed spatial distributions of pesticide inputs, facilitating evaluation of pesticide fate and transport, biogeochemical transformations as well as environmental risk assessment.

## Background & Summary

The application of pesticides is a key element in modern agriculture^1,2^, pesticides residues being ubiquitous in all environmental compartments^3^. Mapping pesticide application rates is relevant for assessing their environmental impact^2,4^, such as, for example, potential effects on soil^4^, surface-subsurface water^5^, and atmospheric drift^6^. Adverse effects of certain pesticides are widely reported in the literature^7,8^. The spatial distribution of pesticides application rates is a critical input required to understand and predict pesticide fate and transport and complex associated biogeochemical transformations, which are typically subject to large uncertainty^9,10^. Yet, application rates can be highly heterogeneous, depending on crop types as well as environmental, climatic, and social drivers. In the European Union (EU) pesticide sales are approximately stable at 350 × 10^3^ tonnes per year^11^. This implies that, on average, 0.08 kg/ha of pesticides are applied to EU soils. Of such huge amount of pesticides, about 82% is degraded biologically in the soil, 10% remains as residue, while about 7% leaches into aquifers^12^. In an effort to control and contain the environmental footprint of agriculture, the European Commission has set a target of reducing pesticide use by 50% within 2030^13^. While the implications of such policy on agricultural EU production would be broad^7^, its implementation is not yet clearly delineated and planned. Official figures reported by EUROSTAT indicate a 4.9% decline in the pesticide sales between 2011 and 2020^11^. At the same time different regulations adopted on the global scale imply that hazards caused by pesticide use may be imported from other countries, where regulations are less restrictive, with EU displaying an import in hazard loads from non-EU countries^14^.

In this scenario, increasing the resolution of data that can be used to assess pesticides contamination pathways and related risks^15^ becomes markedly relevant to further enhance our ability to track the impact of pesticide use on ecosystems.

Maps and spatial datasets have been produced to assess pesticide mass and application rates at the global and continental scale^16,17^. Notably, the PEST-CHEMGRIDS approach^17^ relied on pesticide application rate estimates by crop, which are then tuned using data reported by the United States Geological Survey (USGS). Such estimates were then mapped globally using selected climatic, socio-economic and environmental indicators. Development of remote sensing and data analysis techniques has enhanced our ability to detect cropland extension^18^ and the spatial locations of diverse crop types^19^. Recent products at continental scale feature crop spatial distributions at high resolution^20^. In this work our aim is to leverage the PEST-CHEMGRIDS framework to produce high resolution maps of pesticide application rates across the EU. To this end, we employ three main inputs: i) the PEST-CHEMGRIDSv1.01 maps at coarse scale resolution, ii) high-resolution crop maps from d’Andrimont *et al*.^20^, and iii) the EUROSTAT dataset reporting pesticides for agricultural use. Our data product incorporates uncertainty, that is tracked along the estimation process.

Data are provided as maps of application rates of active ingredients use for different crop types, defined in line with the PEST-CHEMGRIDS approach. Maps are generated for 53 active ingredients across three different pesticides major groups, taking 2018 as the reference year. The produced dataset has a higher spatial resolution than similar currently available products^16,17^. It has relevance for regional studies as it allows direct assessment of pesticide inputs close to landmark and sensitive locations, e.g. cities, rivers, surface water bodies or environmental reserves.

## Methods

**Fig. 1:**
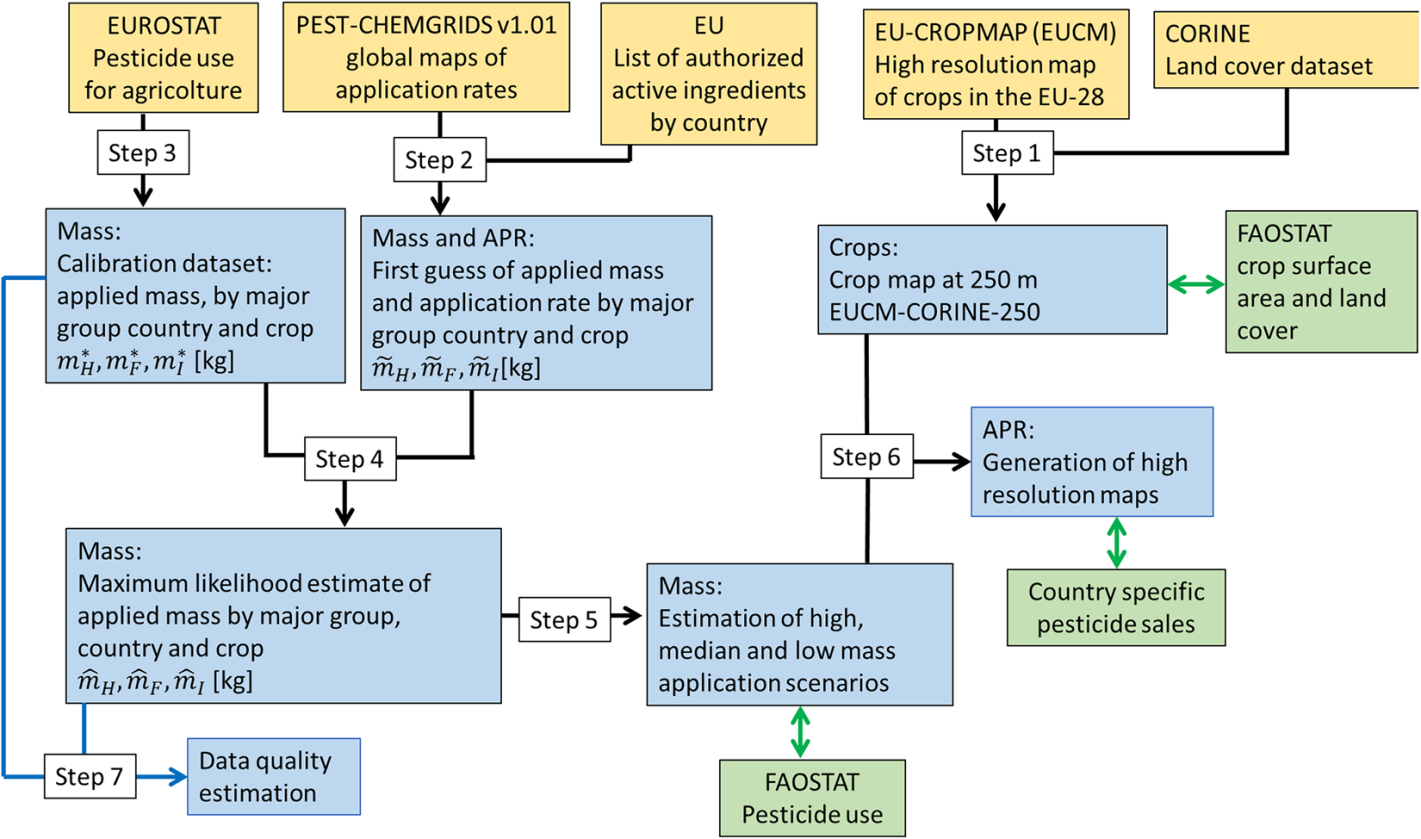
Schematic diagram of the proposed procedure. Yellow boxes indicate input data, blue boxes data analysis steps and green boxes validation data employed. Black and blue lines identify the procedure leading to the estimation of application rate maps (APR) and data quality maps, respectively.

Here, we detail the methodology employed to generate the high-resolution pesticide maps. Our work takes 2018 as the reference year, selected based on the availability of crops spatial mapping data. Figure 1 illustrates the workflow of the implemented methodology while all steps are detailed below.

### Step 1. European crop maps selection and aggregation

The crop maps are required to apply the original PEST-CHEMGRIDSv1.01 framework^17^ and generate high-resolution pesticides application rates maps in EU. The goal of this step is to (re)construct from available sources a spatial dataset featuring the 10 cropping systems in PEST-CHEMGRIDv1.01, i.e. Corn, Soybean, Wheat, Cotton, Rice, Alfalfa, Vegetable and Fruits, Orchard and grapes, Pasture and Hay, and Other crops.

The high-resolution EU crop map (EUCM, d’Andrimont *et al*.^20^) is the key source of information, as it provides crops spatial distribution at 10 m resolution over the whole EU-28 area. The dataset is obtained combining satellite and *in situ* observations of cropping systems through the LUCAS survey. We then manipulated EUCM with two objectives: i) integrating EUCM with the CORINE land cover^21^ dataset to resolve some specific limitations in EUCM that are also acknowledged by the authors^20^, ii) elaborate labels to optimize matching with crop categories that are present in PEST-CHEMGRIDSv1.01. From an operational standpoint, data embedded in EUCM are corrected in the following cases:

- Rice pixels are retrieved from CORINE; thus when a pixel is labelled as “rice” on CORINE, we replace the EUCM value with “rice”.
- While the EUCM label “Woodland and Shrubland (including permanent crops)” contains important information for identifying woody permanent crops, it does not discern other type of vegetation covers. Hence, we selected pixels that are assigned such categories in EUCM and are categorized as “vineyards”, “fruits trees and berry plantations” or “olive groves” on the CORINE land cover dataset. These pixels are classified as “Orchards and Grapes”, consistent with PEST-CHEMGRIDSv1.01.
- All “Grasslands” grid-cells in EUCM that appeared as “Pastures” in CORINE, were eventually considered as “Pastures” in EUCM.

After integration of the two datasets, map resolutions were changed to 250 m, assigning to each pixel in the upscaled map the modal value of the pixels (of 10 m resolution) included in the original map. The upscaling procedure is motivated by our goal of mapping a large number of pesticides application rates. Maintaining a 10 m resolution would complicate data handling without significantly increasing the dataset spatial significance. This map is labeled here as EUCM-CORINE-250.

Map labels are then arranged to match the PEST-CHEMGRIDSv1.01 crop categories as reported in Table 1. The taxonomy of aggregated crop classes resulting from the EUCM-CORINE-250 map is shown in Supplementary Information (Table S2), where entries are gathered from LUCAS and CORINE documentation. The following considerations can be made:

- Crop matching is unambiguous across all datasets for classes Maize, Wheat, Soya and Rice.
- Alfalfa falls into the category Fodder crops in EUCM. We have found no sources to distinguish alfalfa from other fodder crops (cereals and other leguminous). Similarly, Cotton is included in “other industrial crops” in EUCM along with other crop types. Therefore we exclude alfalfa and cotton from the analysis.
- Aggregated classes Vegetable and Fruits and Orchard and Grapes defined by USGS, and used in PEST-CHEMGRIDSv1.01 (see Table 2) are reasonably met in EUCM-CORINE-250.

**Table 1:** Correspondence between EUCM-CORINE-250 and PEST-CHEMGRIDS (PCG in the table) crop classes and percentage of surface area (SA) associated with each label.

EUCM-CORINE-250 value	EUCM-CORINE-250 Legend	PCG equivalent	% of SA
211	Common Wheat	Wheat	22.79
212	Durum Wheat	Wheat	1.37
213	Barley	Other	8.73
214	Rye	Other	1.22
215	Oats	Other	0.34
216	Maize	Corn	15.54
217	Rice	Rice	0.48
218	Triticale	Other	0.02
219	Other cereals	Other	0.01
221	Potatoes	VegFru	0.55
222	Sugar beets	Other	1.24
223	Other root crops	VegFru	0.01
230	Other non-permanent industrial crops	—	0.28
231	Sunflower	Other	3.24
232	Rape and turnip rape	VegFru	5.02
233	Soya	Soybean	0.04
240	Dry pulses	VegFru	1.25
250	Fodder crops	—	1.36
290	Bare arable lands	—	3.13
350	Orchards and grapes	OrcGra	6.27
510	Pastures	PasHay	16.40

**Table 2:** Taxonomy of considered active ingredients, major groups and chemical class definitions (CClass) and names are taken from EUROSTAT.

Major Group	CClass	CClass Name	Active ingredients (Crop)
Herbicides, haulm destructor and moss killer	H01_01	Phenoxy H	2,4-d (ALL), 2,4-db (PH), Dichlorprop (PH), Mcpa (W, PH)
	H02_03	Triazinone H	Metribuzin (S, PH, O)
	H03_01	H based on amide and anilide	Dimethenamid(-p) (C, S, O)
	H03_03	Chloroacetalinide H	Metolachlor(-s) (C, S, VF, PH, O)
	H05_01	Dinitro Aniline H	Pendimethalin (C, S, R, VF, OG, O)
	H06_01	Sulfonylurea H	Halosulfuron (R), Metsulfuron (PH)
	H06_03	Urea H	Diuron (OG, PH)
	H99_01	Aryloxyphenoxy-propionic H	Cyhalofop (R)
	H99_03	Benzoic Acid H	Dicamba (C, S, W, PH, O)
	H99_05	Cyclohexanedione H	Cletodim (S, PH)
	H99_07	Dicarboximide H	Flumioxazyn (S)
	H99_08	Diphenyl ether H	Oxyfluorfen (OG)
	H99_11	Isoxazole H	Isoxaflutole (C)
	H99_13	Nitrile H	Bromoxynil (W, O)
	H99_14	Organophosphorus H	Glufosinate (C, S, OG, PH), Glyphosate (ALL)
	H99_15	Phenylpirazole H	Pinoxaden(W)
	H99_18	Pyridinecarboxylic H	Aminopyralid (PH), Clopyralid (C, W, PH), Picloram (PH)
	H99_19	Pyrodyloxyacetic-acid H	Fluroxypyr (W, PH), Triclopyr (R, PH)
	H99_21	Thiadiazine H	Bentazone (R, VF)
	H99_22	Thiocarbamate H	Tri-allate (W)
	H99_26	Triketone H	Mesotrione (C)
	H99_99	Unclassified H	Clomazone (R)
Fungicides and bactericides	F01_01	Copper Compounds	Copper hydroxide (VF, OG), Copper sulfate (R, OG), Copper sulfate tribasic (OG)
	F01_99	Other inorganic F	Calcium polysulfide (OG)
	F02_03	Dithiocarbamate F	Mancozeb (VF, OG), Ziram (OG)
	F03_01	Benzimidazole F	Thiophanate-methyl (W, VF)
	F04_01	Conazole F	Metconazole (W), Propiconazole (W, R), Prothioconazole (W), Tebuconazole (W)
	F06_01	Microbiological F	Bacillus amyloliquifacien (VF, O)
	F99_03	Anilide F	Flutolanil (O)
	F99_05	Aromatic F	Chlorothalonil (VF, OG, O)
	F99_12	Phthalamide F	Captan (VF, OG)
	F99_16	Strobilurine F	Azoxystrobin (C, W, R), Pyraclostrobin (C, S, W), Trifloxystrobin (R)
Insecticides and acaricides	I01_01	Pyrethroid I	Cyhalothrin-lambda (R)
	I04_01	Organophosphorus I	Chlorpyrifos (C, S, W, OG, O), Dimethoate (W), Ethoprophos (VF)
	I99_08	Nitroguanidine I	Clothianidin (R)
Other PPP	ZR03_99	Other soil sterilant	Metam (VF, O), Metam potassium (VF)

Crop names: C = corn, W = wheat, S = soybean, R = rice, OG = Orchard and grapes, VG = vegetable and fruit, PH = pasture and hay, O = other crops, ALL = all crops. Active ingredient major group names: H = herbicides, F = fungicides, I = insecticides.

Because of the exclusion of two crop types Cotton and Alfalfa, our maps consider eight of the ten classes included in PEST-CHEMGRIDSv1.01 (see Table 1). The EUCM-CORINE-250 map is georeferenced using the EPSG:3035 projected coordinates.

### Step 2. Projection of PEST-CHEMGRIDSv1.01 maps onto fine grid and screening of active ingredients

We perform a projection of PEST-CHEMGRIDSv1.01 maps onto the same georeferenced grid used to map crops in Step 1. For each pixel in EUCM-CORINE-250 we interpolate crop-specific value of application rates provided by PEST-CHEMGRIDSv1.01 using a nearest neighbour approximation. While performing this operation, we apply country-specific pesticides use authorizations and bans for active ingredients as of January 9^th^ 2018. Overall, we obtain a list of 55 active ingredients approved in at least one country. These are categorized following the EUROSTAT^22^ nomenclature, i.e., into ‘major groups’ (e.g., ‘herbicides’, label H) and ‘chemical class’ (e.g., ‘phenoxy herbicides’, label H01_01). Each chemical class contains a variable number of active ingredients (*ai*s). The considered active ingredients pertain to 37 distinct chemical classes and four major groups, as reported in Table 2. Note that in our dataset there are only two active ingredients belonging to the major group “other plant protection product”, hence the active ingredients Metam and Metam potassium are disregarded in this work. In the following, we thus focus on major groups herbicides, haulm destructors and moss killers (H), fungicides and bactericides (F) and insecticides and acaricides (I). These groups are referred to as herbicides, fungicides and insecticides for brevity. All chemical classes and the matching active ingredients included in our dataset are reported for completeness in Table 2, along with the indication of the crop to which they apply in our analysis, this information being retrieved from the PEST-CHEMGRIDv1.01 framework. After projecting the maps onto the refined grid, we obtain spatial values of ${\mathop{APR}\limits^{ \sim }}_{ai}(x,y)$ [kg/ha] which define the application rate of each active ingredient *ai* as well as the total applied mass for each major group per crop and country, ${\widetilde{m}}_{{mg}}({crop},{country})$ [kg], with *mg* = *H*, *F*, *I*.

### Step 3. Preprocessing of calibration data

Here, we assemble a calibration dataset to update the preliminary estimates obtained using PEST-CHEMGRIDv1.01. We collect a calibration dataset from EUROSTAT^22^, which is hereafter denoted as reference dataset. The following steps are performed to obtain a calibration dataset from the reference dataset:

- Data included in the reference dataset are sparse in time; therefore, we consider average quantities from 2015–2020 to obtain reference data to 2018.
- We select applied mass related to chemical classes containing one or more active ingredients included in the PEST-CHEMGRIDSv1.01 dataset, as shown in Table 2.
- The reference dataset indicates crop type, mass, and chemical class of active ingredients. We subdivide data between three different crop classes. We categorize as “Wheat” the applied mass associated with crop labels “Common spring wheat and spelt”, “Common wheat and spelt “, “Common winter wheat and spelt”, “Durum wheat”. We classify as “Corn” the applied mass associated with crops labelled as “Grain maize and corn-cob-mix” and “Green maize”. Composite crop classes are grouped together to avoid possible misclassification due to nonmatching labels. Note that we could exclude data related to “Soybeans” and “Rice”, which represent a very limited portion of crop area in our study. Therefore, these are grouped with other composite crop classes in a “Any Other Crop” (AOC) class.
- We neglect applied mass per country and crop class whose value is smaller than 100 kg.

Upon performing the above steps, we obtain a set of 175 applied mass data, each representing a pesticide mass applied per country, crop and pesticide major group (herbicides, fungicides and insecticides, here labelled as F, H, I). The whole dataset is then split according to the three major groups. We label these data as ${F}_{mg}^{\ast }={m}_{mg}^{\ast }({crop}{,}{country})$ [kg] where *mg* stands for F, H, I, *crop* = [“Corn”, “Wheat”, “AOC”] and *country* indicates the EU-28 Countries (see Supplementary information, Table S1).

**Fig. 2:**
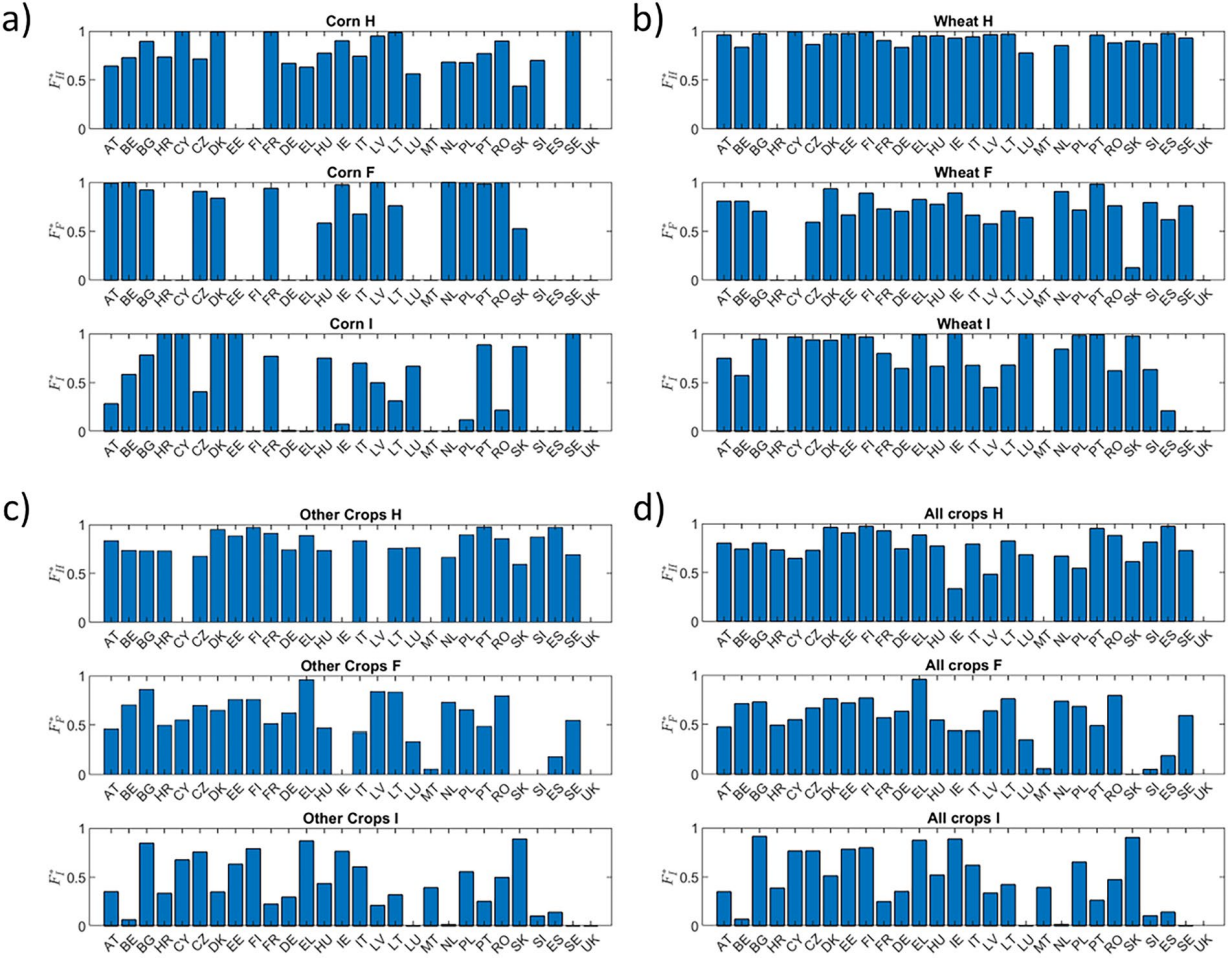
Mass fraction of active ingredients retained in our reference dataset as compared to total mass by major group reported in EUROSTAT for Herbicides (H), Fungicides (F) and Insecticides (I). Results are for (**a**) Corn, (**b**) Wheat, (**c**) Any Other Crop, (**d**) mass-weighted average for all crops.

The selection performed in steps a.-f., described above, implies that our reference dataset includes a subset of the available data reported in EUROSTAT. We quantify here the significance of the considered data over the total figures reported in EUROSTAT for the three considered major groups. Figure 2 displays the ratio ${F}_{mg}^{\ast }={m}_{mg}^{\ast }({crop}{,}{country})/{m}_{mg}^{\ast }({crop}{,}{country})$, where ${m}_{mg}^{\ast }$(*crop,country*) represents the cumulative mass associated with each of the three major groups considered, thus including all chemical classes pertaining to a major group. Note that when ${F}_{mg}^{\ast } > 1$, the value is disregarded. This can happen due to asynchronous reporting of major groups and underlying chemical classes in EUROSTAT. We observe that the major group best represented in our data product is herbicides, followed by fungicides and insecticides. The selected applied mass data ${m}_{mg}^{\ast }$ represent 83.6%, 42.4% and 27.4% of the total herbicide, fungicide and insecticide mass reported in EUROSTAT, respectively. These fractions are labeled as ${\overline{F}}_{H}^{\ast },{\overline{F}}_{F}^{\ast },{\overline{F}}_{I}^{\ast }$ in the following. Considering the mass applied per crop, our dataset includes 82.3%, 81.0% and 48.3% of the total pesticide mass applied for Corn, Wheat and Any Other Crop classes, respectively. We also note that the available data are mostly complete for Wheat, while missing data are relatively more abundant for Corn. The total mass included in our dataset collectively represents 55.4% of the total mass that can be obtained considering the cumulative applied mass of H, F, and I reported in EUROSTAT.

### Step 4. Calibration of applied pesticide mass

Adjustment of the applied pesticides mass by major group is performed assuming the following scaling model based on geographical location and crop type

1 $${m}_{{mg}}={k}_{{mg},C}{\left({crop}\right)k}_{{mg},L}\left({country}\right){\widetilde{m}}_{{mg},{PCG}}{\left({country},{crop}\right)}^{\alpha }$$

where ${\widetilde{m}}_{{mg}}$ is the ‘first guess’ applied mass obtained in step 2. Parameters appearing in (1) are defined as follows

2 $${k}_{{mg},C}\left({crop}\right)=\left\{\begin{array}{ll}{k}_{{mg},{Corn}} & {if\; crop}={corn}\\ {k}_{{mg},{Wheat}} & {if\; crop}={wheat}\\ {k}_{{mg},{AOC}} & {if\; crop}={AOC}\end{array}\right.$$

3 $${k}_{{mg},L}\left({country}\right)=\left\{\begin{array}{cc}{k}_{{mg},{SEU}} & {if\; country}\in {SEU}\\ {k}_{{mg},{CEU}} & {if\; country}\in {CEU}\\ {k}_{{mg},{NEU}} & {if\; country}\in {NEU}\end{array}\right.$$

with

- *SEU* = [Bulgaria, Greece, Spain, France, Italy, Cyprus, Malta, Portugal]
- *CEU* = [Belgium, Czech Republic, Germany, Ireland, Luxembourg, Hungary, Netherlands, Austria, Poland, Romania, Slovenia, Slovakia, United Kingdom]
- *NEU* = [Denmark, Estonia, Latvia, Lithuania, Finland, Sweden]

corresponding to the southern (SEU), central (CEU) and northern (NEU) Europe countries as defined by the relevant EU directive^23^. For each major group, the parameters vector is thus defined as **k**_*mg*_ = [*k*_*mg*_, _*Corn*_,*k*_*mg,Wheat*_, *k*_*mg,AOC*_, *k*_*mg,SEU*_, *k*_*mg,CEU*_, *k*_*mg,NEU*_, *α*]. Parameters are estimated within a maximum likelihood (ML) framework by minimizing the following objective function

4 $$J=\mathop{\sum }\limits_{i=1}^{3}\mathop{\sum }\limits_{j=1}^{28}{({\log }_{10}{m}_{{mg}}^{\ast }({cro}{p}_{i},{countr}{y}_{j})-{\log }_{10}{m}_{{mg}}({cro}{p}_{i},{countr}{y}_{j}))}^{2}$$

Log-transformed values are employed because of the large disparity between applied mass across the dataset. Best estimate of ${{\bf{k}}}_{{mg}}$, ${\hat{{\bf{k}}}}_{{mg}}$, is retrieved by minimizing *J* through the Matlab function *fmincon*. All parameter values are initialized equal to 1, thus assuming ${\widetilde{m}}_{{mg}}$ as a first guess solution. The ML best estimate of *m*_*mg*_ is labelled as ${\hat{m}}_{{mg}}$. Along with parameters best estimates we obtain the minimum value of the negative log-likelihood (NLL), as well as an estimate of the posterior parameter covariance matrix^24^, from which we retrieve the value of the posterior standard deviation, *σ*, for each parameter estimate.

**Fig. 3:**
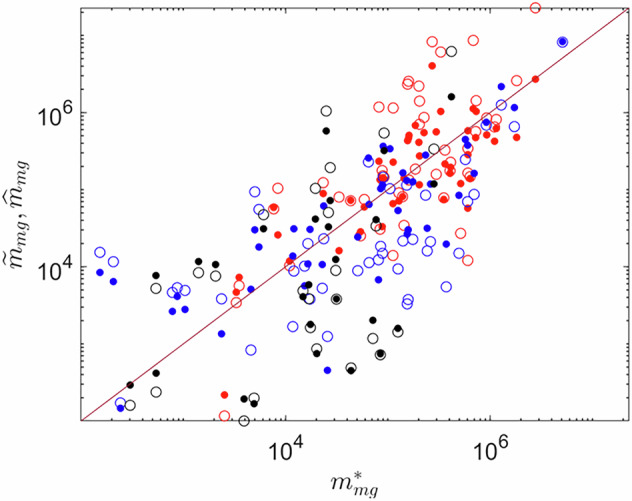
Scatter plot showing the comparison between ${m}_{{mg}}^{\ast }$ (horizontal axis), and ${\widetilde{m}}_{{mg}}$ (empty symbols), ${\hat{m}}_{{mg}}$ (filled symbols) (vertical axis). Results are for ${mg}=H$ (red symbols), ${mg}=F$ (blue symbols), ${mg}=I$ (black symbols), the continuous line indicates the axes bisector.

Calibration results are shown in Fig. 3 and Table 3. The most accurate results are obtained for herbicides, as confirmed by NLL values (lower NLL indicates higher fidelity in reproducing data). This result is consistent with the observation that our dataset provides a relatively high coverage over this class in each country and crop type. Parameter estimation uncertainty is limited for all three major groups, as inferred from the low values of *σ*.

**Table 3:** Calibration results.

mg	NLL	${\hat{{\boldsymbol{k}}}}_{{\boldsymbol{mg}},{\boldsymbol{Co}}}\,{\boldsymbol{\sigma }}({\hat{{\boldsymbol{k}}}}_{{\boldsymbol{mg}},{\boldsymbol{Co}}})$	${\hat{{\boldsymbol{k}}}}_{{\boldsymbol{mg}},{\boldsymbol{Wh}}}\,{\boldsymbol{\sigma }}({\hat{{\boldsymbol{k}}}}_{{\boldsymbol{mg}},{\boldsymbol{Wh}}})$	${\hat{{\boldsymbol{k}}}}_{{\boldsymbol{mg}},{\boldsymbol{SE}}}\,{\boldsymbol{\sigma }}({\hat{{\boldsymbol{k}}}}_{{\boldsymbol{mg}},{\boldsymbol{SE}}})$	${\hat{{\boldsymbol{k}}}}_{{\boldsymbol{mg}},{\boldsymbol{SE}}}\,{\boldsymbol{\sigma }}({\hat{{\boldsymbol{k}}}}_{{\boldsymbol{mg}},{\boldsymbol{SE}}})$	${\hat{{\boldsymbol{k}}}}_{{\boldsymbol{mg}},{\boldsymbol{CE}}}\,\sigma ({\hat{{\boldsymbol{k}}}}_{{\boldsymbol{mg}},{\boldsymbol{CE}}})$	${\hat{{\boldsymbol{k}}}}_{{\boldsymbol{mg}},{\boldsymbol{NEU}}}\,{\boldsymbol{\sigma }}({\hat{{\boldsymbol{k}}}}_{{\boldsymbol{mg}},{\boldsymbol{NE}}})$	$\hat{{\boldsymbol{\alpha }}}\,{\boldsymbol{\sigma }}(\hat{{\boldsymbol{\alpha }}})$
H	87.7	0.542 0.086	1.900 0.310	2.746 0.391	1.065 0.183	1.150 0.178	2.364 0.508	0.906 0.016
F	118.2	0.574 0.135	3.707 0.540	2.353 0.467	0.862 0.280	1.382 0.422	1.326 0.438	0.988 0.027
I	110.2	1.702 0.586	0.721 0.322	1.980 0.836	0.810 0.420	1.168 0.536	0.868 0.364	1.092 0.051

### Step 5. Estimation of uncertainty in the mass of applied pesticides

We generate Monte Carlo realizations of the total mass of applied pesticides. These are obtained upon propagating uncertainty in the parameter estimates to the estimated mass of pesticides. For each major group, we consider the estimated parameters to be independent and normally distributed with mean equal to ${\hat{{\bf{k}}}}_{{mg}}$ and standard deviations as listed in Table 3. The assumption of independence neglects potential correlations between parameters. This is considered as a cautionary measure, as correlation effects may constrain the sampling space limiting sampling to specific portions of the parameter space. The assumption of normality is commonly employed in uncertainty quantification studies when detailed prior information on the statistical distributions of parameters is lacking. We then generate a Monte Carlo sample of 10^3^ realizations of *m*_*mg*_ (*crop,country*) using Eq. (1). From this sample, we denote the first and third quartiles as the low and high estimate of pesticide applied mass, respectively. The median value is also retained, as the nonlinear Eq. (1) may lead to skewed mass distributions. We verified that the sample size yields appropriate estimates of uncertainty bounds, where variations of the related high, low, and median estimate as a function of the number of realizations is below 5%.

### Step 6. Generation of pesticide maps

We calculate the following correcting factors

5 $$\begin{array}{ccc}{K}_{mg,S}=\frac{{m}_{mg,S}}{{\tilde{m}}_{mg}} & with & S=High,Median,Low;mg=H,F,I\end{array}$$

where ${m}_{{mg},S}$ [kg] denotes the applied mass associated with scenario *S* (i.e., high, median, and low estimate of pesticide applied mass). The spatially distributed application rates for all active ingredients are computed applying the correction factors

6 $$\begin{array}{ccc}AP{R}_{ai,S}(x,y)={K}_{mg,S}{\tilde{APR}}_{ai}(x,y) & with & High,Median,Low\end{array}$$

Because each grid cell has a fixed surface area of 6.25 ha in the used projection, the proportionality indices ${K}_{{mg},S}$ obtained from the estimate of applied mass can be directly used to determine the application rates, maintaining the cumulative mass values which is the data employed in the estimation process. Maps are discretized by considering a constant value of ${{APR}}_{{ai},S}$ for each grid cell of the map. All maps are georeferenced in the EPSG:3035 reference system.

### Step 7. Data accuracy assessment

Data accuracy estimation is provided according to the estimation residuals obtained for each active ingredient major group and country, when compared with the calibration dataset (see step 3)^17^

7 $${QI}=1-\frac{|{\hat{m}}_{{mg}}({crop},{country})-{m}_{{mg}}^{\ast }({crop},{country})|}{{\hat{m}}_{{mg}}({crop},{country})+{m}_{{mg}}^{\ast }({crop},{country})}\forall {ai}\in {mg},{mg}=H,F,I$$

The value of *QI* is provided as spatial map using the same grid used to map application rates. When data for a given (*crop,country*) pair is not available we average the indicators obtained for the same country group (NEU, CEU, SEU) for the same crop.

## Data Records

Maps are provided in georeferenced (geotiff) format using EPSG:3035 projected coordinates. For each active ingredient, we include a high (H), median (M), and low (L) estimate map. Data also encompass a spatial accuracy index distribution, which is indicative of the fidelity of the produced maps with respect to our calibration dataset, as reported in Eq. (7). The EUCM-CORINE-250 crop map is also provided, along with the application rate data. All data are provided through figshare repository 10.6084/m9.figshare.27743286^25^.

### Spatial data

**Fig. 4:**
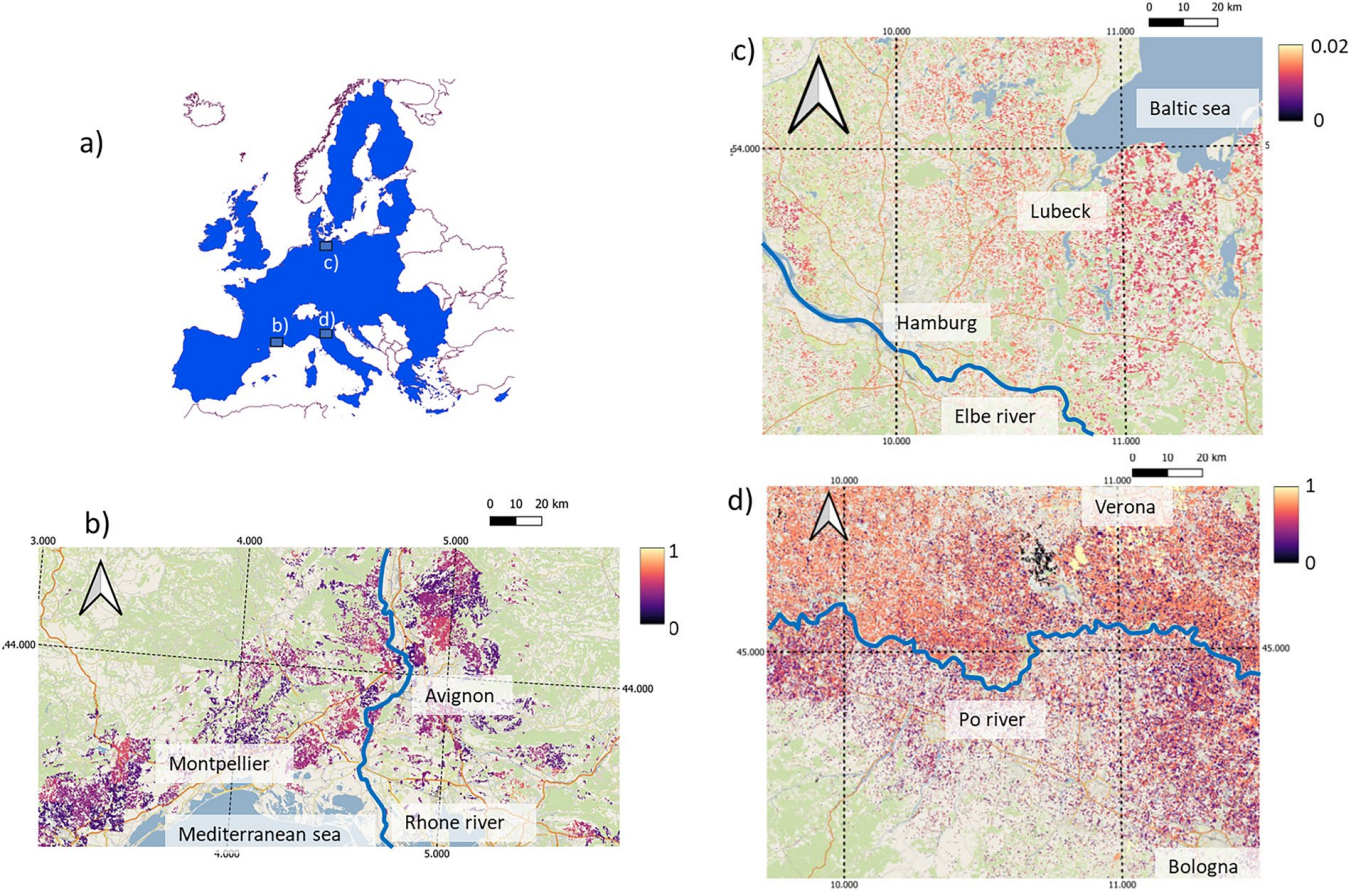
Study area (blue shade in (**a**)) and details of sample maps of median application rates: (**b**) fungicide Mancozeb in southern France, (**c**) insecticide Dimethoate in northern Germany, (**d**) herbicide Glyphosate in northern Italy. All application rates are given in [kg/ha], background information is obtained from Open Street Maps layers, labels on maps boundaries indicate longitude and latitude (WGS84 coordinates).

Illustrative spatial maps are depicted in Fig. 4, showing the spatial distribution of the application rate of three selected pesticides in three distinct areas, which discerns local features such as rivers, natural reserves, and urban areas. These maps can be used to inform environmental assessments across various scales and environmental compartments. The original PEST-CHEMGRIDSv1.01 would not allow regional or local analyses because of the coarse scale resolution of the dataset. Our data can fill this gap upon providing an opportunity to analyze spatial proximity between relevant landscape features (rivers, water bodies, cities) and the estimated pesticide loads.

Our dataset aims at improving our spatial understanding pesticides application at the local scale. We faced several challenges to produce the dataset. These are mainly related to limited availability of spatially explicit information, which ultimately has an impact on the released data. We list here specific limitations related to the data released:

- Released maps do not account for specific agricultural practices, such as organic farming, that can influence pesticide use at the local scale. While aggregated figures of organic farming are available from EUROSTAT, the spatial distribution of these has not been documented at European level.
- We employ a static crop map, where each spatial location is assigned to a fixed crop type. This assumption neglects double cropping which will likely impact pesticide use.
- The data can serve as an element to evaluate the impact of specific policies at the local scale. To this end, our data should be integrated with field data and campaigns to assess the actual concentrations in the environment. These can be highly variable given the wide set of process that can influence pesticide transport across environmental compartments.
- Quality of data is variable by crop class. Data associated with wheat crops generally show better accuracy than those related to corn. For other crops, application rates are aggregated across a vast set of crops (the AOC class). This is necessary here because of difficulties in matching crop classes between diverse datasets. This should be considered when employing the maps.
- Our maps do not include all pesticides authorized in EU, thus cannot be used for a comprehensive evaluation of the total pesticide load, yet they can be used for assessing environmental impact of pesticide use by crop and for the specific active ingredients considered in our analysis.

## Technical Validation

### Validation of EUCM-CORINE-250 crop map

The EUCM-CORINE-250 map is compared against two FAOSTAT domains, i.e. the harvested areas of specific crop types by country^26^ and the total temporary cropland area^27^. As harvested areas include double cropping it is in principle not directly comparable to our crop map where one pixel is assigned to a single crop type and cropping cycle. However, this comparison provides crop-specific reference numbers.

**Fig. 5:**
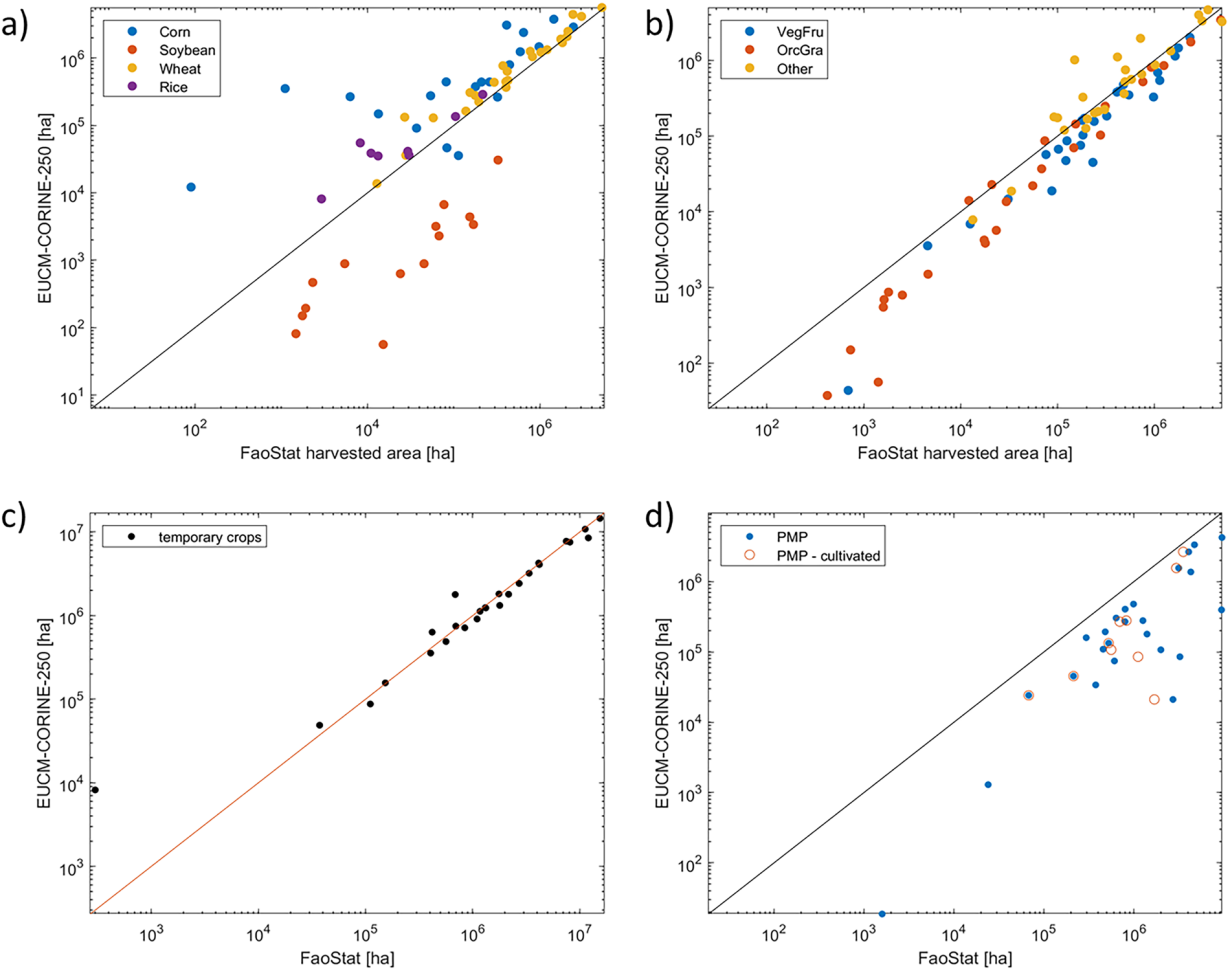
Scatterplot of estimated crop areas obtained from EUCM-CORINE-250 against (**a**) harvested areas reported in FAOSTAT for 2018 for single crops classes, (**b**) harvested areas reported in FAOSTAT for 2018 for aggregated crops classes, (**c**) crop (land cover) areas reported for all temporary crops, (**d**) permanent meadows and pasture (PMP) land cover areas. In (**c**) the EUCM-CORINE-250 data cumulatively consider the classes Wheat, Soybean, Corn, Rice, VegFru and Others.

Figure 5a presents the crop-by-crop analysis for the four single crop systems for which pesticides map are generated, i.e., Corn, Wheat, Soya, Rice. The EUCM-CORINE-250 estimation provides the best performance for wheat, whose result align with the reference for all countries. Surface areas estimates assigned to the class Rice appear reasonable when compared with FAOSTAT, indicating that data fusion between EUCM and CORINE provided an improvement over the original EUCM map (see discussion in d’Andrimont *et al*.^20^). Soybean surface crop and Corn crop areas are underestimated and overestimated by EUCM-CORINE-250, respectively. This result is likely due to double cropping, where Soybean is often combined with wheat or corn crops. The EUCM-CORINE-250 assigns a single label to a pixel, thus neglecting that the same crop surface area may be utilized twice per year and for different crops. Corn crop area displays important deviations in Scandinavian countries, where EUCM-CORINE-250 vastly overestimates the FAOSTAT data. This result may be explained upon observing that FAOSTAT does not report corn for fodder. Results are more closely aligned in southern Europe. Figure 5b shows the assessment of EUCM-CORINE-250 against FAOSTAT for aggregated crop classes. The three classes display good agreement with aggregated FAOSTAT data. VegFru and OrcGra show slight underestimation of harvested areas. The good agreement found for OrcGra class supports our choice to integrate CORINE data into EUCM to detect the presence of this particular crop class.

Figure 5c displays a comparison of the total aggregated crop surface considering all classes [Wheat, Corn, Rice, Soya, VegFru, Other] and the temporary crop land cover data reported by FAOSTAT for each country. Note that the latter refers to crop area and not harvested area, unlike in Fig. 5a,b. The results closely align, thus showing a good agreement between temporary crops area and the aggregated results of the EUCM-CORINE-250 map. This result also confirms that mismatches in corn crop areas (Fig. 5a) are likely due to different aggregations used for this class, as discussed above. Finally, Fig. 5d presents the comparison between surface area labelled as ‘Pasture and Hay’ and the land use classifications ‘Permanent Pasture and Meadows (PMP)’ and ‘PMP – cultivated’ from FAOSTAT. These comparisons are selected as, in principle, the CORINE pasture class description aligns better with the PMP class in FAOSTAT. Results show that EUCM-250-CORINE generally underestimates pasture area compared to FAOSTAT, probably because of the general difficulty in discerning natural from managed pastures from satellite images. Quantitative indicators supporting this discussion are reported in Supplementary information (SI) Table S3-4.

### Validation of pesticide applied mass by major group and crop

**Fig. 6:**
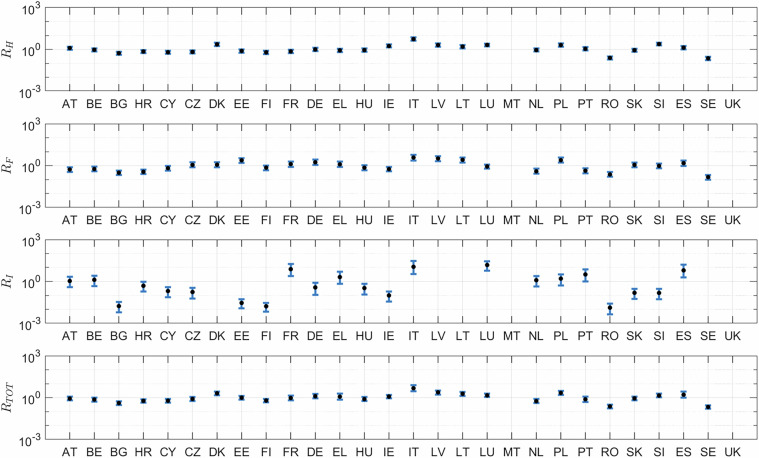
Values of ${R}_{{mg}}$ by country for herbicides, ${mg}=H$, fungicides, ${mg}=F$, insecticides ${mg}=I$ and all major groups combined, ${mg}={TOT}$. Bars indicate the range of variation between high and low cumulative mass estimates, black dots indicate the median value.

We tested whether the estimated mass of active ingredients obtained from the calibration process is in line with the calibration dataset, ${M}_{mg}^{\ast }$, by computing

8 $${R}_{{mg}}=\frac{{m}_{{mg},S}({crop},{country})}{{m}_{{mg}}^{\ast }({crop},{country})},S={Low},{Median},{High}$$

this ratio being ideally equal to 1. Results are depicted in Fig. 6, where the low, high and median estimates for each country and major group are shown. We observe that for fungicides and herbicides deviation of *m*_*mg,Median*_ from the reference data is typically within one order of magnitude. For insecticides uncertainty is generally larger and accuracy is lower, in line with the observation that this class is less represented in our calibration dataset.

**Fig. 7:**
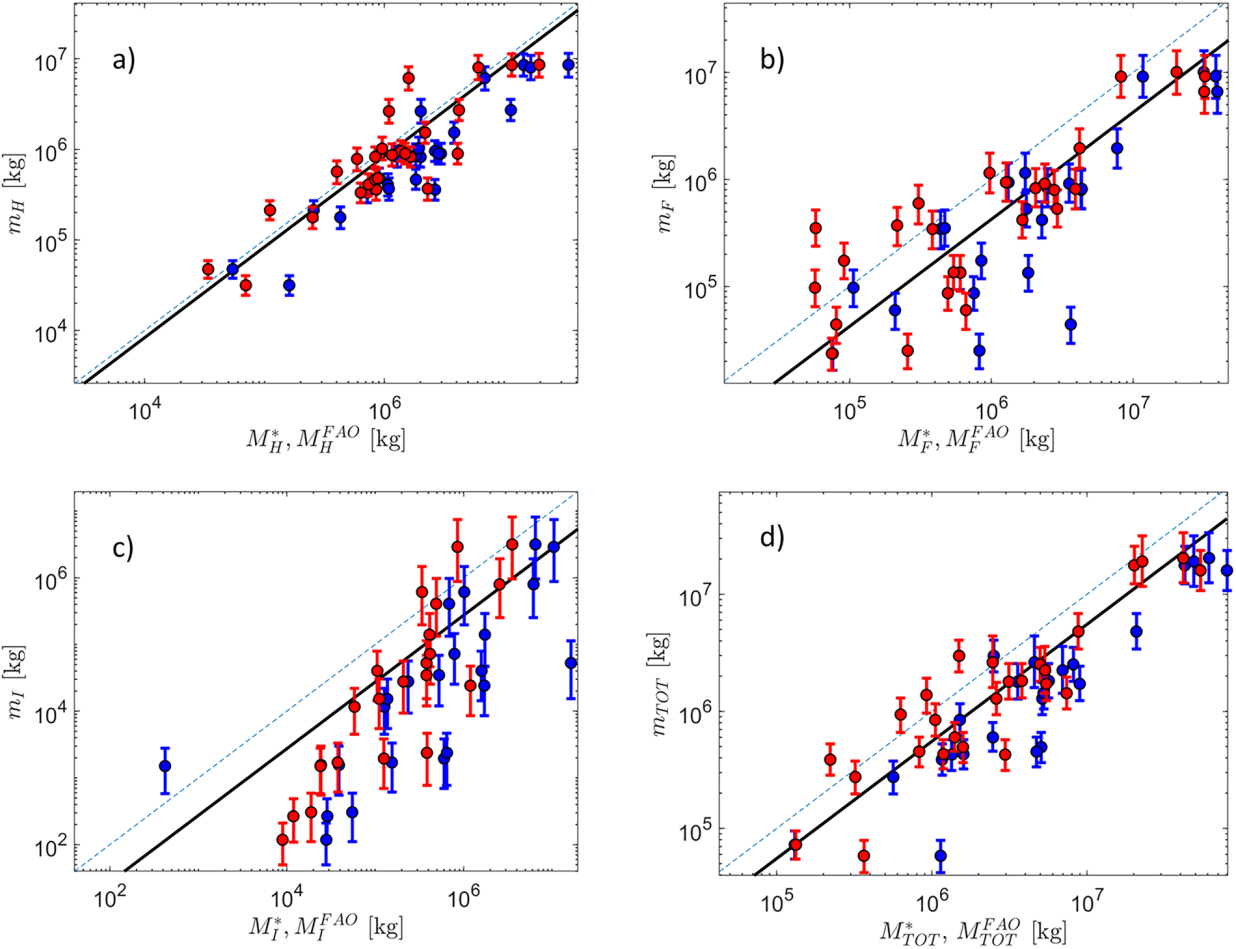
Scatter plot comparing the predicted cumulative mass of pesticides, *M*_*mg*_, and that reported in EUROSTAT, ${M}_{mg}^{\ast }$, (in red) and in FAOSTAT, ${M}_{{mg}}^{{FAO}}$ (in blue) dataset. Results are for (**a**) herbicides, (**b**) fungicides, (**c**) insecticides, (**d**) total. In each subplot, the solid black line indicates the reference value, ${F}_{mg}^{\ast }\times {M}_{mg}^{\ast }$, the dashed blue line indicates the axes bisector.

Figure 7 reports a comparison between our predictions of the cumulative mass, ${M}_{{mg}}$, and the EUROSTAT and FAOSTAT^28^ counterparts for pesticide major groups. Because our dataset includes only a fraction of the total mass per major group, Fig. 6a–d also shows ${m}_{mg}^{\ast }\times {\overline{F}}_{mg}^{\ast }$ (see black lines), i.e. the reference value that can be expected from our results on average for a specific major group. For instance, ${\overline{F}}_{mg}^{\ast }=0.836$ for herbicides (mg = H). Hence, we expect that estimated mass should reproduce on average this fraction with respect to the figures reported by EUROSTAT. The dispersion of the points about the reference line and the uncertainty in the estimates is the lowest for herbicides and the highest for insecticides, while fungicides display intermediate performance (see also Supplementary Information, Tables S5, 6).

**Fig. 8:**
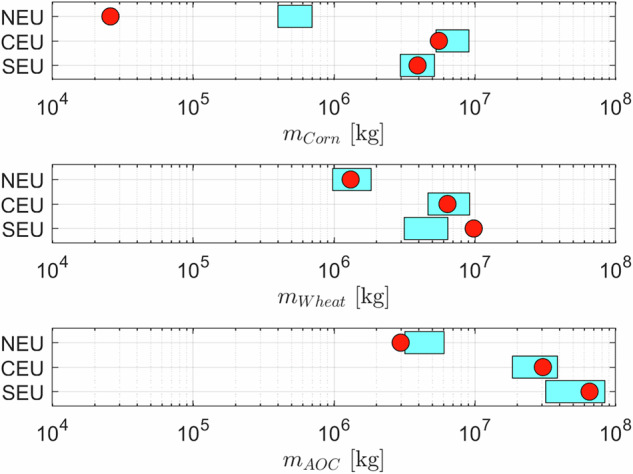
Comparison between crop specific mass of applied pesticide retrieved by EUROSTAT (red points) and the range between high and low predictions (cyan filled rectangles). Data are presented for SEU, CEU, NEU countries.

Figure 8 provides a comparison of pesticides applied by crop, considering the data from SEU, CEU, NEU. Masses applied to Wheat and Corn are of the same order of magnitude for CEU and SEU, our estimates providing an underestimation of pesticides applied to wheat in SEU. In NEU countries, our predictions overestimate the mass of pesticides applied to corn, which is still negligible compared to what is applied to wheat and AOC. EUROSTAT cumulative mass applied to the AOC class is in line with our estimates.

### Validation of pesticide applied mass by active ingredient

**Fig. 9:**
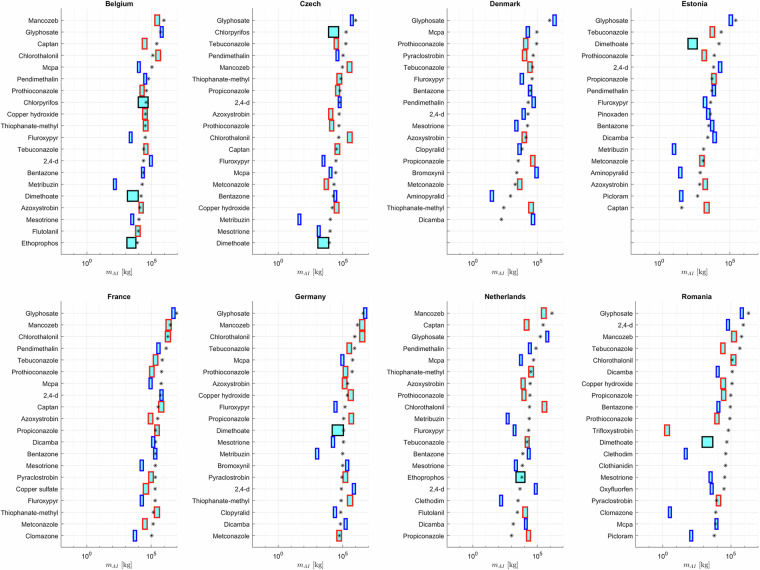
Comparison between country specific sales data (black asterisks) for active ingredients and our predictions, shown by cyan filled rectangles identifying high and low estimates. Blue, red and black rectangles indicate herbicides, fungicides and insecticides, respectively.

We validate our results using data on pesticide sales or use reported by individual countries by individual active ingredient, *ai*. Data are gathered for Belgium^29^, Czechia^30^, Denmark^31^, Estonia^32^, France^33^, Germany^34^, Netherlands^35^ and Romania^36^. Results are reported in Fig. 9, for the most employed 20 active ingredients for each country. Among the considered countries, data available for France, Belgium and Germany align most closely with our predictions. Larger deviations can be seen when considering results associated with Netherlands and Romania (see also Supplementary information, Table S7). Diverse performances obtained by countries may also be due to different policies.

Note that two *ais* that are typically found among the most sold are Mancozeb and Glyphosate. For these two ais our predictions are in line with reported figures in most countries.

## Supplementary information

Supplementary Information

## Data Availability

All analyses are performed using Matlab R2023b. Codes and data to reproduce our data are available through figshare repository^25^.
