# Supplementary material for: Pesticides application rate maps in the European Union at a 250 m spatial resolution
Source: Sci Data. 2025 May 1;12:725. doi: 10.1038/s41597-025-05031-7 (PMC12045998; doi:10.1038/s41597-025-05031-7)
Supplement: Supplementary file 1 — Supplementary Information [file 41597_2025_5031_MOESM1_ESM.pdf]

Supplementary information for

Pesticides application rate maps in the European Union at a 250 m spatial resolution

Authors

G.M. Porta[1], L. Casse[2], A. Manzoni[1], M. Riva[1], F. Maggi[2], A. Guadagnini[1]

Affiliations

[1]Department of Civil and Environmental Engineering, Politecnico di Milano, Piazza Leonardo da Vinci 32, 20133, Milano, Italy

[2]Environmental Engineering, School of Civil Engineering, The University of Sydney, Sydney, New South Wales, Australia

Calibration data

Table S1 lists calibration data obtained as described in Step 3 of the data processing procedure. Note that figures included here refer to the reported mass used in each country only considering the chemical classes reported in Table 3 of the manuscript. Zero values indicate missing data. The dataset is most complete for Herbicides major group and Wheat crop. Data associated with 'Corn' are generally sparser as compared to 'Wheat' and 'AOC' particularly when Fungicides and Insecticides are considered. Data sparsity may be due to a combination of these three factors: *i*) data is not reported in the EUROSTAT reference dataset, *ii*) a given crop ('Wheat' or 'Corn') is not present in one country thus pesticide use is not reported, *iii*) active ingredients from the chemical classes considered in our study are not employed in given country. Our procedure estimates applied mass by aggregating countries across three regions (Northern, Central and Southern Europe), thus alleviating possible issues related to unreported data for a given country.

Table S1. Mass [kg] of applied pesticides pertaining to considered chemical classes (see Table 3 in the main article body) by country and crop type.

	Herbicides			Fungicides			Insecticides		
**Country**	**Corn**	**Wheat**	**AOC**	**Corn**	**Wheat**	**AOC**	**Corn**	**Wheat**	**AOC**
AT	159394	91568	410886	15344	85032	697220	3927	2068	31435
BE	201019	89609	729886	153	175268	1284320	302	1409	24956
BG	203085	128207	361501	2364	372069	64602	3094	84466	27314
HR	112655	0	352197	0	0	242466	6448	0	17497
CY	0	7483	55576.5	0	0	152841	0	1002	22946
CZ	203961	405432	606157	1039	261003	572612	4097	30922	125283
DK	54037	604390	393402	873	157987	138682	0	5541	5443
EE	2527	83924	143942	0	17326	23581	0	4919	4375
FI	0	112156	606041	0	11767	50764	0	375	6798
FR	2773337	6102751	8215770	4997	2583269	9040709	20167	22334	237689
DE	2307899	2113231	4127299	0	1447692	3811138	0	54526	87781
EL	33311	58840	925352	0	12047	917054	10355	14803	282122
HU	667385	237616	790285	11469	450471	830809	66107	22351	128549
IE	18823	66343	704887	453	127448	279528	0	11945	8636
IT	683500.5	225683.5	271079	5488.5	104760	5098149	43630.5	6085	418079
LV	3297	157116	170292	0	88622	51075	0	1199	2091
LT	7651	184267	298406	243	140301	89953	0	4716	5551
LU	8400	3515	10940	0	4585	22850	0	0	0
MT	0	0	0	0	0	5173	0	0	0
NL	155938	43255	802324	207	65507	1976081	0	540	19236
PL	328506	721365	2184520	16530	602205	2385255	542	76676	245346
PT	87948	16692	804911	25659	1206	1876087	5690	1098	122147
RO	1815757	637725	1148484	80918	495015	1725499	18817	71328	91851
SK	81794	138783	319513	783	125643	184028	7136	16839	76464
SI	37882	5987	43737	0	7209	176269	0	102	1941
ES	0	1105203	4824891	0	233784.8	5881492	0	26247	483824.2
SE	1552	514904	1206125	0	154594	249538	0	0	6389
UK	0	0	0	0	0	0	0	0	0

Taxonomy of crop aggregated classes

Table S2. (see following page) Taxonomy of aggregate classes as taken from EUCM-CORINE-250 labels and related entries from LUCAS and CORINE. VegFru and OrchGra stand for ‘Vegetable and Fruits’ and ‘Orchard and Grapes’. In column ‘source label’ L stands for LUCAS, C stands for CORINE. Iems with an asterisk are matched in the corresponding group as labelled in USGS/PNSP dataset, NSE stands for not specified elsewhere.

PEST-CHEMGRIDv1.01 category	EUCM-CORINE-250 label	source labels	Taxonomy
VegFru	221 Potatoes	L-B21	Potatoes*
	223 Other root crops	L- B23	Fodder beet*, Fodder kale*, Swedes, Carrots*, Turnips*, Jerusalem artichoke, Sweet potatoes*, Fodder parsnips, Yam, Manioc, Cassava, Horseradish*, Tuberous chervil, Other tropical tubers, Garlic*, Onion*, Radishes*, Red beet*, Shallots, Mangolds and beet, other root or tuber crops NSE
	232 Rape and turnip rape	L- B32	Turnip Rape, Rape
	240 Dry pulses	L- B41	Dry peas*, Garden peas*, Chickpea, Garbazo Bean*, Cowpea, Pigeon Pea*, Field peas*, Field beans*, Lentils, Lentil vetches, Vetches, Lupins, Peanuts
		L-B42	Tomatoes*
		L-B43	Cabbages*, Cauliflowers*, Artichokes*, Asparagus*, Cardoons, Celery*, Chicory*, Cress, Endives, Garden orache, Leeks, Lettuce*, Rhubarb*, Purslane, Spinach*, Rocket, Watercress, Green bean*, Green Peas*, Melons, Cucumber, Eggplants*, Okras*, Peppers*, Pumpkins*, Watermelons*, Cultivated mushrooms, Wild products, Other fresh vegetables NSE
		L-B44	Flowers and ornamental plants, Bulbs and tubers, cut-flowers, Cut- leafage
		L-B45	Strawberries*
OrchGra	350 Orchard and grapes	C-221	vine plants*
		C-222	berry shrubs: black and/or red currants, raspberries, gooseberries, blackberry;<br>orchards: apples*, pears*, plums*, apricots*, peaches*, cherries*, quinces, other rosaceae and figs*;<br>citrus species*: oranges*, lemons*, mandarins, tangerines*, grape fruits*, pomelos*;<br>nut crops: chestnut*, walnut*, almond*, hazelnut*, pistacia*; tropical fruit species: avocados, bananas, guavas, mango*, kiwis*, passionfruits, papayas*, pineapples, pomegranates, brazil nuts, cashew nuts, coconuts, nutmegs;<br>industrial plants: coffee, cacao, mulberry, tea
		C- 223	Olive groves*
Other	213	L-B13	Barley*
	214	L-B14	Rye*
	215	L-B15	Oats*
	218	L-B18	Triticale*
	219	L-B19	Sorghum*, Buckwheat, Millet, Canary grass, Quinoa, Fonio, other cereals NSE*
	222	L-B22	Sugar beets*
	231	L-B31	Sunflower*

Quantitative distance indicators

We provide here quantitative distance indicators between our datasets and the validation datasets, as indicated in Figure 1 of the manuscript. We provide the following quantitative distance indicators, which are employed considering our estimates (x_e) and the related reference datasets (x_{ref})

$$NRMSE = \frac{\sqrt{\frac{1}{N_D}\sum_{i=1}^{N_D}\left(x_{e,i}-x_{ref,i}\right)^2}}{\mu(x_{ref})} \quad \text{(S1)}$$

$$R = \frac{1}{N_D-1}\sum_{i=1}^{N_D}\left(\frac{x_{e,i}-\mu(x_{pred})}{\sigma(x_{pred})}\right)\left(\frac{x_{e,i}-\mu(x_{ref})}{\sigma(x_{ref})}\right) \quad \text{(S2)}$$

$$\beta = \frac{\mu(x_e)}{\mu(x_{ref})} \quad \text{(S3)}$$

$$\alpha = \frac{\sigma(x_e)}{\sigma(x_{ref})} \quad \text{(S4)}$$

where $\mu(\cdot)$,$\sigma(\cdot)$ indicate sample mean and standard deviation and N_D is sample size. These indicators include classical distance measures, i.e., the normalized root mean squared distance (NMRSE) and the Pearson correlation coefficient (R).

We also provide the Kling-Gupta efficiency[1] (KGE) as a summary metric based on the above indicators in (S2)-(S4)

$$KGE = 1 - \sqrt{(1-R)^2 + (1-\alpha)^2 + (1-\beta)^2} \quad \text{(S5)}$$

Note that all indicators all computed for positive data in the reference datasets, thus omitting missing data.

Table S3 and S4 refer to results obtained for crop surface areas against the two reference FAOSTAT[2,3] datasets.

Table S5 and S6 consider aggregated Herbicide, Fungicides and Insecticides (H, F, I) and aggregated figures (TOT) considering these three major groups. Table S5 refers to the EUROSTAT[4] dataset, employed for calibration, while Table S6 reports distance indicators obtained when comparing our estimates to FAOSTAT[5]. Note that for computing distance measures the reference data are scaled by the fractions $\bar{F}_H^*, \bar{F}_F^*, \bar{F}_I^*, \bar{F}_{TOT}^*$. Finally, Table S7 lists distance measures obtained when considering national level sales figures obtained for nine selected countries. Distance measures in Tables S5-7 refer to both linear and log-transformed data.

Table S3. Distance indicators between crop surface area in EUCM-CORINE-250 and FAOSTAT harvested areas.

Crop type[3]	NRMSD	R	β	α	KGE
Corn	2.225	0.783	2.265	1.913	-0.576
Soybean	1.533	0.889	0.057	0.086	-0.318
Wheat	0.495	0.965	1.248	1.208	0.674
Rice	0.668	0.986	1.531	1.242	0.416
VegFru	0.484	0.965	0.698	0.798	0.635
OrcGra	0.731	0.999	0.716	0.716	0.598
Other	0.635	0.904	1.143	1.026	0.826

Table S4. Distance indicators between crop surface area in EUCM-CORINE-250 and FAOSTAT data. Estimated area for Temporary crops in EUCM-CORINE-230 include cumulative surface areas Corn, Wheat, Soya, Vegetable and Fruits and Other classes.

Land Use[2]	NRMSD	R	β	α	KGE
Temporary Crops	0.259	0.987	1.071	1.107	0.871
Permanent meadows and pastures	1.173	0.696	0.316	0.427	0.058
Perm. meadows & pastures - Cultivated	0.709	0.904	0.424	0.751	0.365

Table S5. Distance indicators evaluated between predicted applied mass and data reported in EUROSTAT, results are reported considering reference data and our best estimate values in linear and logarithmic scale.

	Linear data					Log-transformed data				
	NRMSD	R	β	α	KGE	NRMSD	R	β	α	KGE
H	0.976	0.814	0.868	0.735	0.651	0.049	0.886	0.992	0.975	0.883
F	1.033	0.850	0.913	0.812	0.744	0.079	0.840	1.010	0.928	0.824
I	5.156	0.726	2.595	3.674	-2.126	0.173	0.877	0.920	1.748	0.238
TOT	0.829	0.877	0.945	0.863	0.809	0.047	0.902	1.001	0.984	0.901

Table S6. Distance indicators evaluated between predicted applied mass and data reported in FAOSTAT, results are reported considering reference data and our best estimate values in linear and logarithmic scale.

	Linear data					Log-transformed data				
	NRMSD	R	β	α	KGE	NRMSD	R	β	α	KGE
H	1.188	0.862	0.484	0.418	0.210	0.049	0.886	0.992	0.975	0.883
F	0.982	0.879	0.662	0.644	0.494	0.079	0.840	1.010	0.928	0.824
I	1.659	0.511	0.611	0.783	0.338	0.173	0.877	0.920	1.748	0.238
TOT	0.937	0.935	0.558	0.546	0.363	0.060	0.915	0.958	1.032	0.900

Table S7. Distance indicators evaluated between predicted applied mass for individual active ingredients and reported sales in national datasets, results are reported considering data and median applied mass in linear and logarithmic scale.

	Linear data					Log-transformed data				
	NRMSD	R	β	α	KGE	NRMSD	R	β	α	KGE
BE[6]	2.816	0.697	1.697	1.647	0.002	0.218	0.693	1.132	0.632	0.503
CZ[7]	2.480	0.738	1.506	1.626	0.154	0.244	0.670	1.119	0.786	0.589
DK[8]	2.170	0.989	0.532	0.437	0.268	0.256	0.355	0.985	0.869	0.342
EE[9]	3.528	0.981	2.081	2.477	-0.830	0.316	0.508	1.149	0.642	0.374
FR[10]	1.820	0.966	1.543	1.634	0.164	0.327	0.739	1.154	0.449	0.372
DE[11]	1.324	0.908	0.661	0.624	0.485	0.202	0.733	1.052	0.562	0.484
NL[12]	4.962	0.440	1.803	2.169	-0.524	0.248	0.504	1.020	1.221	0.456
RO[13]	9.239	0.923	5.512	3.988	-4.412	0.899	0.139	1.380	0.572	-0.034

References

1. Gupta, H. V. & Kling, H. On typical range, sensitivity, and normalization of Mean Squared Error and Nash-Sutcliffe Efficiency type metrics. *Water Resources Research* **47**, 2011WR010962 (2011).
2. FAO. FAOSTAT Land, Inputs and Sustainability: Land Use. https://www.fao.org/faostat/en/#data/RL (2024).
3. FAO. FAO: FAOSTAT Production: Crops and Livestock products. https://www.fao.org/faostat/en/#data/QCL (2024).
4. EUROSTAT. *Agri-Environmental Indicator - Consumption of Pesticides*. https://ec.europa.eu/eurostat/statistics-explained/index.php?title=Agri-environmental_indicator_-_consumption_of_pesticides (2023).
5. FAO. FAO: FAOSTAT Pesticides Use. https://www.fao.org/faostat/en/#data/RP (2024).
6. SPF, S. P., Sécurité de la Chaîne Alimentaire et Environnement Service Produits phytopharmaceutiques et Fertilisants. Données de vente. https://fytoweb.be/fr/plan-de-reduction/vigilance/donnees-de-vente (2024).
7. Ukzuz. Statistika uvádění účinných látek obsažených v přípravcích na ochranu rostlin na trh. https://eagri.cz/public/portal/ukzuz/pripravky-na-or/ucinne-latky-v-por-statistika-spotreba/statistika-uvadeni-ul-por-na-trh (2024).
8. Ministry of Environment of Denmark, Environment Protection Agency. Annual Pesticide statistics (in danish). https://eng.mst.dk/chemicals/pesticides/pesticides-statistics/agriculture-etc (2024).
9. Statistics Estonia. KK2085: SALES OF PESTICIDES BY ACTIVE SUBSTANCE. https://andmed.stat.ee/en/stat/keskkond__pollumajanduskeskkond/KK2085 (2024).
10. OFB, Office Francaise de la biodiversite. Descriptif des donnes de ventes de produits pytopharmaceutiques. (2023).
11. Federal Office of Consumer Protection and Food Safety, Germany. Domestic sales and export of plant protection products and their active substances. https://www.bvl.bund.de/EN/Tasks/04_Plant_protection_products/01_ppp_tasks/02_ppp_AuthorisationReviewActSub/03_ppp_DomesticSalesExport/PPP_domesticSales_and_Export_node.html (2023).

12. Statistics Netherlands. Gewasbeschermingsmiddelen landbouw. https://opendata.cbs.nl/statline/portal.html?_la=nl&_catalog=CBS&tableId=85130NED&_theme=229 (2024).
13. National Institute for statistics, Romania. Pesticides on the market. https://insse.ro/cms/en/tags/pesticides-market (2024).
